# Sexual dysfunction in patients with polycystic ovary syndrome and its affected domains

**Published:** 2014-08

**Authors:** Tahereh Eftekhar, Farnaz Sohrabvand, Neda Zabandan, Mamak Shariat, Fedyeh Haghollahi, Akram Ghahghaei-Nezamabadi

**Affiliations:** 1 *Vali-e-Asr Reproductive Health Research Center, Tehran University of Medical Sciences, Tehran, Iran.*; 2 *Departments of Obstetrics and Gynecology, Vali-e-Asr Hospital, Tehran University of Medical Sciences, Tehran, Iran.*; 3 *Maternal-Fetal and Neonatal Health Research Center, Tehran University of Medical Sciences, Tehran, Iran.*

**Keywords:** *Polycystic ovary syndrome*, *Physiological sexual dysfunction*, *Infertility*, *Psychologicalsexualdysfunction*, *Libido*, *Arousal*.

## Abstract

**Background:** Polycystic Ovary Syndrome (PCOS) is presented with characteristic complications such as chronic an ovulation, obesity, and hyperandrogenism which can affect sexual function in women of reproductive age.

**Objective:** Herein we evaluated the frequency and predisposing factors of sexual dysfunction in infertile PCOS patients.

**Materials and Methods: **In this cross-sectional study, 130 married women with a definite diagnosis of PCOS who were referred due to infertility were recruited. They were evaluated concerning their sexual function in the domains of desire, arousal, lubrication, orgasm, satisfaction and pain with the female sexual function index (FSFI) questionnaire.

**Results: **The frequency of sexual dysfunction was verified 57.7% in PCOS patients with the domains of desire and arousal being commonly affected in 99.2% and 98.5%of cases respectively. BMI had a significant effect on sexual desire and arousal (p=0.02) while the effect of hirsutism was significant on all domains (p<0.001 for total FSFI score) except for dyspareunia.

**Conclusion:** PCOS patients markedly suffer from sexual dysfunction as comorbidity. It seems appropriate to screen all PCOS patients for sexual function with a simple short questionnaire such as FSFI. Targeted interventions could be considered to help improve their quality of life along with other treatments.

## Introduction

Polycystic ovary syndrome (PCOS) is the most common endocrine disorder in women of reproductive age. The estimate prevalence is 5-24% in different populations (1, 2). PCOS is characterized by large ovaries, menstrual irregularities, clinical and biochemical hyperandrogensim. It is associated with obesity, insulin resistance, lipid disorders, an ovulatory infertility and endometrial cancer (1, 3). There are several studies assessing the impact of symptoms and treatment of PCOS patients on their life quality (1, 4-6). Hirsutism, acne, alopecia and infertility can lead to diminished "feminine identity" and psychological stress in these patients (1, 7-9). Women with PCOS are at an increased risk for depression and anxiety disorders (1, 8, 9). Several studies have revealed diminished quality of life (QOL) in PCOS patients. Women with PCOS and their partners are less satisfied with their sex life (4, 7, 10, 11).

Alterations in the physical and aesthetic standard (hirsutism, obesity, acne, and alopecia) and an imbalance of sexual hormones are consequently observed, which can lead to a loss of quality of life and to the sexuality of the patients, a greater prevalence of mood disorders, such as major depression and bipolar disorder (, , ). Both the mood disorders and their medicinal treatments are deleterious for the sexual function (, ). The greater part of the studies specifically directed to the evaluation of the sexuality of patients with PCOS refers to the psychosexuality or to sexual orientation (18-20). Studies that go profoundly into the sexual function of patients with PCOS were rare. Changes in physical appearance associated with PCOS may lead to decreased sexual satisfaction (21). Therefore, we studied the associations between Body Mass Index (BMI), and hirsutism on sexual functioning in this population of PCOs infertile women.

A sexual problem, or sexual dysfunction, refers to a problem during any phase of the sexual response cycle that prevents the individual or couple from experiencing satisfaction from the sexual activity and resulting from physical, social, and psychological factors (22). "Epidemiological studies in the United States have estimated that Female sexual dysfunction (FSD) affected 43% of women in the general population over the past 12 months" (23). "In the United Kingdom, 5.8% of women have reported recent sexual dysfunction, and 15.5% have reported lifelong sexual dysfunction" (24). The rate of FSD for middle aged women in Latin America; it is approximately 58% (25). For the first time we evaluated the FSFI in Iranian PCOS patients.

The sexual function index (SFI) questionnaire measures the sexual function in women. It assesses specific domains of sexual functioning including desire, sexual arousal, lubrication, orgasm, satisfaction and pain (26). In view of multiple factors that can impair the sexual function of these patients, it would seem essential to evaluate the importance of this problem and the main factors related to it. With this purpose, a study was set up, to investigate the sexual function of patients with PCOS. Furthermore, the main clinical characteristics potentially related with sexual dysfunction (SDy) were also investigated. Herein we evaluated the FSFI for the first time in such patients. We also investigated the possible associated factors in PCOS with different domains of sexual functioning.

## Materials and methods

This cross-sectional study was conducted at the Infertility Department of Obstetrics and Gyneocoloy Ward in Vali-e-Asr Hospital in Tehran university of Medical Sciences from March 2009- April 2011. A total of 130 infertile married women with definite diagnosis of PCOS, according to Rotterdam criteria, were recruited after obtaining a written consent. This study was confirmed by the Ethics Review Committee of Tehran University of Medical Sciences. The following participants were excluded: those with diabetes mellitus, degenerative illnesses, other endocrinopathies; those with illnesses that could cause a cycle of menstrual disorders; those who had used hormones up to 60 days before the selection process; and patients with a diagnosis of primary amenorrhea.

Sexual function was assessed using the FSFI questionnaire in Persian as previously translated and validated (27). Sexual dysfunction was assessed using Female Sexual Function Index (FSFI) scale (27-29). The scale is a 19-item questionnaire, developed as multidimensional self-report instrument for the assessment of the key dimensions of sexual functioning in women in last month.

This questionnaire consists of questions in six domains including desire, arousal, lubrication, orgasm, satisfaction and pain that scored by patients self-reported. The items of the scale are divided into six domains which include desire (2 questions), subjective arousal (4 questions), lubrication (4 questions), orgasm (3 questions), satisfaction (3 questions) and pain (3 questions). Libido (sexual desire) or interest is a feeling that includes wanting to have a sexual experience, feeling receptive to a partner's sexual initiation, and thinking or fantasizing about having sex, Arousal (desire for sexual activity with sexual stimulations), Orgasm (to reach orgasm after adequate sexual arousal and stimulation), Dysparunia (pain in the pelvis or vagina during any stage of normal sexual stage). "The total FSFI score is the sum of all scores obtained in each five domain .The higher score, is the better in the sexuality. The score of 26.55 was considered as the cut-off value for diagnosis of female sexual dysfunction" (30).

Cronbach’s alpha coefficient was calculated to evaluate reliability of questionnaire and it was 0.816. The primary outcome measure in this study was to assess sexual function in PCOS patients. Additional outcomes of interest were to investigate sexual function of PCOS patients in relation to their age, BMI, menstrual pattern, degree of hirsutism (according to Ferriman- Gallway Scoring system) and past obstetric history. In all patients with PCOS and idiopathic hirsutism physical examinations and tests were be done by gynecologist and other causes including hyper-androgenic, congenital adrenal hyperplasia, cushing, hyper prolactinemia and hypo thyroidism or tumors secreting androgens were excluded.

The degree of hirsutism was assessed using the FG scoring system (Ferriman-Gallwey score), each individual body area (body areas including the lip, chin, chest, upper abdomen, lower abdomen, upper arm, forearm, thigh) is visually scored by patients self-reported on a scale of 0-4, where 0 indicates no terminal hair growth and 4 indicates full male-pattern terminal hair growth. A score ≥6-8 generally defines hirsutism (31). The definitions for the various BMI categories were normal (<25), and obese (>25).

Statistical analysis

Data were analyzed with SPSS software version 17 (SPSS Inc. Chicago, IL, USA) P≤0.05 was considered significant. Data are expressed as mean±SD and percentages. Average and SD were used to evaluate descriptive data. χ^2^ test was used to compare categorical variables, and Student t- test and analysis of variance were used to compare the continuous variables (FSFI variable). Bivariate correlations were investigated by Pearson product-moment correlation coefficient. 

## Results

Women in ambulatory accompaniment for PCOS (n=130) were sequentially evaluated. Mean age of patients was 27.02±4.27 years ranging from 19 to 38. Mean BMI was 26.98±3.84 kg/m^2^ ranging from 19.38 to 37. Seventy (53.8%) of them had education levels higher than high school, and 123 (94.6%) were housewives. Among the patients with history of live birth 8 (44.4%) had natural vaginal delivery (NVD) and 10 (55.6%) had cesarean section (CS). The majority of the patients had irregularities in their menstrual pattern. The mean Ferriman-Gallway Score (FGS) was 8.00±3.27 ranging from 3 to 21. The frequency of different PCOS symptoms in patients is given in table I.

In our study, mean FSFI score was 25.93±3.92 (CI 95%: 25.26-26.65, range: 10.3-34.5). If the score of 26.55 was considered as the cut-off value for diagnosis of female sexual dysfunction, the prevalence of sexual dysfunction in PCOS patients was 57.7%, n=75 (20). FSFI scoring (mean & median level) in different domains is reported in table II. There was not any relationship between age and FSFI in different domains (p=0.4). Patients who education levels were higher than high school had significantly better sexual function than patients with lower education, total FSFI score of 26.62 vs. 25.17 respectively (p=0.04). Women with higher education specifically showed significant higher FSFI scores in orgasm, lubrication and arousal (p=0.03, p=0.002 and p=0.02 respectively). There was not any relationship between type of last delivery and total score of FSFI in patients with live birth history. 

However, dysfunction in lubrication in patients with NVD compared with patients with CS is greater but the differences was not statistically significant. Total FSFI score did not show any significant difference in women with higher than normal BMI levels (p=0.09). Normal BMI than higher levels had significant better scores on desire (4.26±0.01 vs. 3.66±0.77, p=0.001) and on satisfaction (5.10±0.15 vs. 4.69±0.05, p=0.001). Hirsutism (FGS >8) had significant negative effect on total FSFI score (p<0.001) in all different domains. If the FGS score of 6 and higher was considered the cut-off value to diagnose hirsutism, there was still a difference between hirsute and non-hirsute patients for total FSFI score (p<0.001). In other words, the score of hirsutism had reverse correlation with FSFI.

**Table I:** Demographic characteristics and frequency of PCOs symptoms

		**N (%)**
Education		
	Senior	60 (46.2)
	Junior	70 (53.8 )
Job		
	Housewife	123 (94.6)
	Employee	7 (5.4)
Delivery Type		
	NVD:	8 (44.4)
	C/S	10 (55.6)
Sexual Disorder		
	Yes	75 (57.7)
	No	55 (42.3)
Oligomenorrhea		119 (91.3)
Amenorrhea		6 (4.6)
History of live birth		18 (13.8)
History of abortion		30 (23.1)
Normal, BMI <25		64 (49.2)
Obesity, BMI >25		20 (15)
Morbid Obesity, BMI >30		6 (4.6)
Hirsutism, FGS >8		72 (55.4)
Hirsutism, FGS >6		92 (70.8)
Age*		27.02 ± 4.27
BMI*		26.98 ± 8.4
FSFI*		25.93 ± 3.92

BMI: Body Mass Index

FGS: Ferriman-Gallway score

T-test

Chi-square test

* are presented as mean±SD

**Table II:** Domains of sexual dysfunction in infertile PCOS patients according to female sexual function index (FSFI)

	**FSFI score (mean** **±SD)** *	**Median**	**Prevalence of dysfunction (%)** **
Desire	3.78 ± 0.88	4.28	99.2
Arousal	3.94 ± 0.84	5.08	98.5
Lubrication	4.53 ± 0.3	5.45	90.2
Orgasm	4.45 ± 0.08	5.05	86.9
Satisfaction	4.71 ± 0.9	5.04	78.5
Pain	4.53 ± 1.02	5.51	80.0

* T-test

** Chi-square test

**Table III:** Relationship between FSFI score and factors in PCOS patients

**Factors**		**FSFI score (mean±SD)**	**p-value**
Mean age**		25.93 ± 3.92	0.45
Education*			
	Lower than high school	26.62 ± 0.72	0.004
	Higher than high school	25.17 ± 0.58	
BMI**		25.93 ± 3.92	0.09
Type of Delivery *			0.06
	NVD	25.45 ± 0.53	
	C/S	25.86 ± 0.41	
Hirsutism **			0.01
	FGS>8	25.50 ± 0.69	
	FGS<8	27.0 ± 0.81	

* T-test

** Correlation testMultiple Regression Test

## Discussion

We evaluated sexual function of infertile PCOS women with FSFI questionnaire. FSFI had been previously used to assess sexual functioning in several diseases (32-35). According to our results there is a high prevalence of sexual dysfunction among PCOS patients associated with lower education levels, and hirsutism. BMI levels higher than normal had decreased desire and satisfaction. Sexual dysfunction has been reported to be 10-50% in different Iranian populations (36, 37). According to our results PCOS patients reveal one of the highest rates (57.7%). Previously, sexual dysfunction is reported to be 13.3% among PCOS patients using the Arisona Sexual Experience Scale (ASEX) (38). Sexual function preservation in the aforementioned study could be due to lower average age of patients who were included from a completely different population. The ASEX scale only consists of 5 questions and may not investigate sexual function as comprehensive as FSFI.

Other Studies have revealed a prevalence of 57.4%, 32.6% and 33.8% for sexual dysfunction in women with multiple sclerosis, diabetes and metabolic syndrome respectively (29, 32, 33). Demographic factors such as age, job and education are found to have effect on sexual function in different studies (36, 39, 40). In our study, the range of patients’ age was limited (19-34 yrs) and we could not find any relationship between age and sexual dysfunction. However, higher education levels improved sexual functioning in different domains. The frequency of sexual dysfunction in the domain of desire was the highest (99.2%) and the domain of satisfaction showed the lowest frequency (78.5%). Satisfaction specifically is reported to be lower in PCOS patients than normal women (7, 41).

In normal Indian women, FSFI domain scores suggested difficulties with desire in 77.2%; arousal in 91.3%; lubrication in 96.6%; orgasm in 86.6%, satisfaction in 81.2%, and pain in 64.4% (42). Also In normal Korean women, FSD was detected as a desire problem in 44.0% of women, an arousal problem in 49.0%, a lubrication problem in 37.0%, an orgasm problem in 32.0%, a satisfaction problem in 37.0%, and a pain problem in 34.6% (43). In Indian women FSFI total scores suggested FSD in two-thirds of the 149 women (73.2%; 95% CI: 65.5-79.6%) (43). Infertility is one of the most stressing factors in women`s life and it may influence their satisfaction and quality of life (44, 45). In our study all the patients were infertile which may have impaired their sexual function that could justify the high prevalence we found. The majority of our patients had menstrual irregularities which reveals their hormonal disturbances. Such abnormalities are highly associated with sexual dysfunction (38).

In the study of Khademi *et al* in Iranian population with using the Sexual Function Questionnaire (SFQ) to assess FSD, was reported that only 7out of 100 infertile Iranian women reported normal sexual functioning. The most prevalent sexual problem among these women was decreased sexual arousal (80%) Tayeb *et al* in 2009 has reported that the most common sexual problems in infertile Iranian females were anorgasmia (83.7%) and decreased libido (80.7%) and indicated the sexual desire and frequency of coitus in infertile women has reduced significantly after infertility diagnosis (46, 47). Jain and associates have indicated that sexual problems in infertile women is consisted of dyspareunia, decreased libido, and orgasmic failure were the most common problems in their study 

The results of Ramezani study on four normal women living in urban areas provinces showed that sexual dysfunction is prevalent among Iranian women and who consider attractive wife, seems less impaired sexual function (49). Major depressive disorder is characterized by loss of interest, reduction in energy, lowered self-esteem, inability to experience pleasure, this constellation of symptoms may be expected to produce difficulty in sexual relationship and depressed patients have shown sexual dysfunction 2-3 times more than non-depressed individuals (50). It has also been found that PCOS women experience less sexual attractiveness and sexual desire (51).

Other factors such as high BMI, and hirsutism can affect one`s perception of sexual attractiveness (7). In this study, BMI did not have any significant effect on the total sexual function score. However, increasing BMI levels resulted in diminished score on desire and satisfaction domains. Ferraresi showed that the PCOS obese women were at a higher risk for sexual dysfunction and lower FSFI scores, and women with borderline FSFI values, regardless of their obesity status (52). Stovall study showed that increasing BMI was associated with a significant reduction in the orgasm subdomain (21).

In Stovall study no significant associations were found in regard to hirsutism but the negative impact of hirsutism on sexual function and quality of life in PCOS patients has been widely assessed in different studies (2, 21). We observed very same outcomes in our study. The high frequency of dysfunction in desire domain could mainly be due to hirsutism and high BMI since they are considered to affect women’s body image. Women may also experience emotional states such as depression, anxiety, and lowered self-esteem that are known causative factors of sexual dysfunction. Marital distress may arise following the diagnosis of infertility, and women who have had multiple, unsuccessful treatment attempts are known to be at a greater risk of psychological distress (51, 53, 54). These studies suggest that psychological factors and partner relationships are important factors formative of sexual function. Thus, a two-group study to demonstrate the relationship between sexual dysfunction to social and psychological factors, are necessary.

One limitation of this study is the recruitment population. Since we included patients from a tertiary center who had encounters with other gynecologists, they might have higher rates of comorbidities and sexual dysfunction. There could be a limitation to extend our results to fertile PCOS patients who have less contact with health care system. Due to possible role of PCOS, we did not exclude patients with previous psychological findings including anxiety disorders and depression which are common in PCOS. In general, sexual dysfunction could be considered as comorbidity in PCOS patients. Desire is the most impaired domain in their sexual functioning which is highly correlated with hirsutism that mostly affects patients’ body image. Affected women could be referred for a consult with psychologist or sexologist who could improve their quality of life with simple interventions. Prospective clinical studies are suggested to evaluate possible targeted treatments in order to regain normal sexual function in PCOS patients.

## Footnote

***This article extracted from M.D. thesis. (Neda Zabandan)***
